# Hyperspectral Sensing for Turbid Water Quality Monitoring in Freshwater Rivers: Empirical Relationship between Reflectance and Turbidity and Total Solids

**DOI:** 10.3390/s141222670

**Published:** 2014-11-28

**Authors:** Jiunn-Lin Wu, Chung-Ru Ho, Chia-Ching Huang, Arun Lal Srivastav, Jing-Hua Tzeng, Yao-Tung Lin

**Affiliations:** 1 Department of Computer Science and Engineering, National Chung Hsing University, 250 Kuo Kuang Rd., Taichung 402, Taiwan; E-Mail: jlwu@dragon.nchu.edu.tw; 2 Department of Marine Environmental Informatics, National Taiwan Ocean University, 2 Pei-Ning Rd., Keelung 202, Taiwan; E-Mail: b0211@mail.ntou.edu.tw; 3 Department of Soil and Environment Sciences, National Chung Hsing University, 250 Kuo Kuang Rd., Taichung 402, Taiwan; E-Mails: j65k3@hotmail.com (C.-C.H.); arunitbhu2009@gmail.com (A.L.S.); hua7526@smail.nchu.edu.tw (J.-H.T.)

**Keywords:** hyperspectral sensing, multiple-regression, neural network, remote sensing, total solids, turbidity, turbid water

## Abstract

Total suspended solid (TSS) is an important water quality parameter. This study was conducted to test the feasibility of the band combination of hyperspectral sensing for inland turbid water monitoring in Taiwan. The field spectral reflectance in the Wu river basin of Taiwan was measured with a spectroradiometer; the water samples were collected from the different sites of the Wu river basin and some water quality parameters were analyzed on the sites (*in situ*) as well as brought to the laboratory for further analysis. To obtain the data set for this study, 160 *in situ* sample observations were carried out during campaigns from August to December, 2005. The water quality results were correlated with the reflectivity to determine the spectral characteristics and their relationship with turbidity and TSS. Furthermore, multiple-regression (MR) and artificial neural network (ANN) were used to model the transformation function between TSS concentration and turbidity levels of stream water, and the radiance measured by the spectroradiometer. The value of the turbidity and TSS correlation coefficient was 0.766, which implies that turbidity is significantly related to TSS in the Wu river basin. The results indicated that TSS and turbidity are positively correlated in a significant way across the entire spectrum, when TSS concentration and turbidity levels were under 800 mg·L^−1^ and 600 NTU, respectively. Optimal wavelengths for the measurements of TSS and turbidity are found in the 700 and 900 nm range, respectively. Based on the results, better accuracy was obtained only when the ranges of turbidity and TSS concentration were less than 800 mg·L^−1^ and less than 600 NTU, respectively and used rather than using whole dataset (R^2^ = 0.93 *versus* 0.88 for turbidity and R^2^ = 0.83 *versus* 0.58 for TSS). On the other hand, the ANN approach can improve the TSS retrieval using MR. The accuracy of TSS estimation applying ANN (R^2^ = 0.66) was better than with the MR approach (R^2^ = 0.58), as expected due to the nonlinear nature of the transformation model.

## 1. Introduction

Rain is the major cause of soil erosion which brings sediment to the stream network. Soil erosion alone causes damages equal to around $6 billion annually in the United States [1]. Total suspended solid (TSS) in the inland surface water, such as streams, lakes, and reservoirs, is one of the most visible indicators of erosion or related problems worldwide [2–6]. Large concentration of TSS can damage hydroelectric power and pumping equipment, increase the cost of water treatment, reduce the useful storage capacity, minimize the flood control capability of reservoirs, decrease the light penetration, adversely affects fish and shellfish populations, spawning grounds and alter the aquatic food chain [7]. In rivers, various types of organic pollutants are also transported along with suspended solids [8,9], making TSS useful as a tracer for water quality dynamics and the distribution of pollutants as well [10,11]. The TSS levels of aquatic reservoirs also play an important role in water quality management since they is directly related to the total primary productivity, transport of contaminants such as heavy metals and organics as well as to define the survival quality of aquatic organisms [12]. Turbidity is a physical water quality parameter, which measures the light penetration capacity to the depth of any water reservoir [13]. TSS or turbidity levels of any water body provide quantitative information as to the state of water quality [14]. Therefore, it is useful to monitor and assess the TSS concentration and turbidity levels of inland waters. Density, shape, size, and nature of the particles and color of water govern the affinity between TSS and turbidity [15]. It has been reported that one NTU of turbidity is equvalent to around 1–2 mg/L of TSS [16]. Regression curves, optical properties of water and sediment mass transfer corelations can be obtained at the same time through TSS and turbidity level measurements [17–19]. However, synoptic information on TSS (or turbidity) at a regular frequency is difficult to obtain from the routine *in situ* monitoring network because TSS is a temporal and spatially heterogeneous parameter [20]. Currently, measurements of TSS and turbidity of surface water are based on *in situ* measurements and subsequent laboratory analyses. Traditional approaches are time-consuming, discrete in time, require space and do not easily lend themselves to understand the temporal and spatial dimensions of TSS of surface water which contributes toward more understanding about the water quality [21–23]. Hence, there is necessity to develop reliable, spatially covering and cost-efficient monitoring techniques that can be deployed easily, and which should be capable of monitoring surface water quality in a synoptic view. The potential for assessing surface water quality from reflected solar radiation through remote sensing has already been recognized [24–26]. According to Santini *et al*. [27] various types of arial sensors as well as satellites have been used during the last three decades to collect the information about aquatic organisms. Ocean water quality assessment using remote sensing data has been carried out since the deployment of the first remote sensing satellite Landsat-MSS (Multi-spectral Scanner) [28]. The concentrations of optically active water constituents (*i.e.*, TSS and turbidity) can also be estimated using remote sensing approaches by interpreting the received radiance at different wavelengths [29]. While remote sensing has been used extensively for both marine and coastal waters [26,30,31], the application of remote sensing to inland surface waters is constrained by the need of high spatial resolution image data and thus remains limited by spectral resolution capabilities. Commonly, open ocean waters are naturally oligotrophic, whereas fresh waters have higher TSS concentrations. Doxaran *et al.* [32] determined the composition of water in terms of turbidity using visible and near-infrared (NIR) wavelength satellite data. Teodoro *et al.* [33] also studied the TSS concentration in sea water using multispectral satellite data. Analyses have proven a non-linear correlation for TSS concentration and sea water reflectivity. When applying an artificial neural network, ASTER, HRVIR, and TM sensors performed better than ASTER and HRVIR sensors in the estimation of TSS using visible and near-infrared band images. Olmanson *et al.* [34] used airborne hyperspectral remote sensing to study about the water quality parameteres of the Mississippi river and its tributaries in Minnesota. Because, very high concentration of TSS was observed in the river water perhaps due to the massive soil erosion phenomenon [35]. Around the world, agricultral, drinking as well as industrial needs depend on the use of inland surface water reservoirs [36,37]. In turbid inland waters, the fluctuation of suspended matter, dissolved organic carbon (DOC), and phytoplankton make it difficult to apply universal remote sensing models for predicting water quality as compared to open ocean waters. For this reason, many site-specific models have been developed using ground-truth data from a variety of environmental settings [38–41]. However, whilst some encouraging experimental results have been observed in presence of only low TSS concentrations (less than 50 mg·L^−1^). But, those calibrated models are applicable only for the inland water bodies which have low levels of turbidity [42]. Papoutsa *et al.* [43] assessed the levels of turbidity in inland water bodies using Landsat TM/ETM+ and CHRIS/PROBA spectral regions through field spectroscopy. *In situ* spectroradiometric measurements, Secchi disk depth and turbidity measurements were carried out during the study of Asprokremmos Reservoir in Paphos District, Cyprus. Among applied several regression analyses, Landsat TM/ETM+ Band 3 (R^2^ = 0.85) and CHRIS/PROBA Bands A30 to A32 (R^2^ = 0.90) have shown the highest correlation. Landslides and debris flows is common due to heavy typhoon-season rainstorm and frequent earthquakes in Taiwan. Around 921 earthquakes (7.3 magnitude) occurred on September 21, 1999 which brought voluminous suspended solid from landslides and the debris flows to the streams. Therefore, additional *in situ* samples and reference field spectra for the higher TSS concentrations of surface water are important in Taiwan.

This research examines the spectral reflectance of stream water characterized by heterogeneous TSS concentration and turbidity levels and aimed to identify an appropriate data analysis approach which could aid the quantification of TSS or turbidity at high concentrations using modern remote sensing data. In the present study, the spectral signatures of water reflectance were measured using a portable spectroradiometer, together with ground-truth measurements of TSS concentration, level of turbidity, and chlorophyll, for selected sampling locations in the Wu River basin, Taiwan. The characterization of inland surface waters for higher TSS concentration assisted in the interpretation of inland water quality parameters using remote sensing imagery technique during this study. Both multiple-regression and neural network methods were used to model the transformation function between the TSS and turbidity in stream water and the radiance measured at the spectroadiometer.

## 2. Study Site

**Figure 1.**
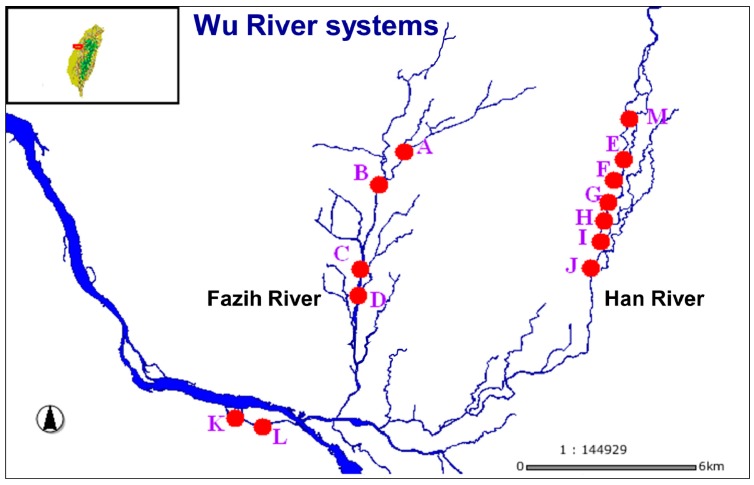
The study area. Red symbols are the locations of water quality sampling stations and referent features in the Wu River systems.

Study sites were selected to cover a wide variety of optically different waters in central Taiwan where the surface water samples were characterized by high turbidity, with average Secchi disc values generally lower than 0.5 m. The chosen sites were highly polluted with no bottom brightness. The sampling points were distributed along the tributaries, namely, Fazih River, Han River, and Dali River in the Wu River basin. The TSS spatially and temporally was generally high in all the seasons, and also highly heterogeneous, with average values varying from 100 to 700 mg·L^−1^. To cover the wide range of water quality variation, the sampling points were placed along the main axis of Wu River basin located between the longitude of 120.628273 E ∼120.707225 E and the latitude of 24.106496 N ∼ 24.177789 E (Figure 1).

## 3. Methods

### 3.1. Field Work

Field optical reflectance measurements were carried out with a portable spectroradiometer (ASD FieldSpec Dual UV/VNIR, Boulder, CO, USA) at a wavelength range of 350–1050 nm with 1.4 nm nominal bandwidth, against a halon white reference panel. The data were taken above the water surface in vertical downward direction. Two of these instruments were used: one collecting the upwelling radiance from the water and the other one collecting downwelling irradiance. Upwelling radiances from the water body were retrieved with approximately 100% reflectance across the entire spectrum. The reflectance is the ratio of the upwelling radiance emanating from the water surface to downwelling irradiance. Ten separate sets of scans of each sample were measured and an average value was taken for final analysis. Only the 400–920 nm portion of the spectrum was collected due to the observed low signal-to-noise ratio over water at both ultraviolet and near-infrared wavelengths. The effect of atmospheric dispersion and light absorption were considered negligible as the distance of the spectroradiometer from the water surface was less than 2 m. Surface water samples were undertaken in the vertical direction and collected near the surface (50 cm depth) with a 5 L polyethylene bucket immediately after spectrum measurements. Surface water samples were collected and stored in 2.5 L dark polyethylene bottles for further analysis. The samples were stored in ice during transportation and subsequently filtered for chlorophyll-a (Chl-a), *i.e.*, within 4 h. In addition, auxiliary water quality parameters (*i.e.*, turbidity, temperature, pH, and dissolved oxygen) were measured *in-situ* at each station, after which transparency of water was also measured by using Secchi disk. The locations of sampling points are shown in Figure 1. Field data, consisting of 160 samples, were collected between August and December, 2005.

### 3.2. Laboratory Methodology

In the laboratory, water samples were stored in black PE bottles at 4 °C before analysis. The concentration of Chl-a was determined within 24 h, whereas COD and TSS were analyzed within one week. Analyses of TSS, turbidity, Chl-a and COD levels in water samples were done as per the Standard Methods for the Examination of Water and Wastewater (APHA) [44]. Concentration of Chl-a was measured at 663 and 670 nm using fluorescence (Hitachi 650-40, Tokyo. Japan) at 663 nm emission wavelength and 670 nm excitation wavelength. A HI93703 turbidimeter (Hanna, Woonsocket, RI, USA) was used to measure turbidity (NTU) at each level of TSS. For COD determination, samples were refluxed in acid solution with a strong oxidizing agent like potassium dichromate (K_2_Cr_2_O_7_). After digestion, the remaining K_2_Cr_2_O_7_ was back titrated with ferrous ammonium sulfate to determine the amount of K_2_Cr_2_O_7_ consumed and the oxidizable matter is calculated in terms of oxygen equivalent.

## 4. Data Analysis

### 4.1. Laboratory Methodology

Regression analysis has been a traditional empirical method of choice for modeling transfer functions [29,45–48]. At the optical wavelengths, passive remote sensing signals are affected by the volume scattering and the surface reflection. It is possible to develop the methods for the estimation of water quality variables using optical observations. Single band data has widely been used in the study of water quality.

However, attempts have been made to find combinations of more wavelengths which would provide more information about water quality parameters as compared to the single band. In this study, multiple-regression (MR) retrieval algorithms were employed for water quality variables using hyperspectral sensor data, and which can be expressed as:

(1) $$D_{\text{turbifity or TSS}}=A_{0}+\sum_{i=400}^{920}A_{i}\left(R_{i}\right)$$

where *D_turbidity_* or *D_TSS_* is the water quality, e.g., turbidity or TSS, *R_i_* is the reflectance at wavelength *i*. *A_0_* and *A_i_* are constants.

### 4.2. Artificial Neural Network

To compare the regression analysis with standard methods of water quality estimation, an artificial neural network (ANN) was also applied to the data. ANN was originally developed to model the functions of the human brain and has been successfully applied in the fields of classification, pattern recognition, and signal processing. ANN is a non-linear computational system that models the network of neurons in the brain to perform problem-solving tasks. In many applications, the performance of ANNs has been found to be superior to classical statistical approaches, and they have been widely used as pattern classifiers or estimators in many fields. The advantage of this approach is providing good results while dealing with problems where there are complex relationships between inputs and outputs, and where input data may be distorted by high noise levels. Neural networks imitate the structure of the biological nervous system. It consists of a set of nodes; input nodes will receive the input signals, output nodes provide the output signals and unlimited numbers of intermediate layers contain the intermediate nodes. There are various different neural network architectures and they utilize different learning algorithms to perform tasks.

**Figure 2.**
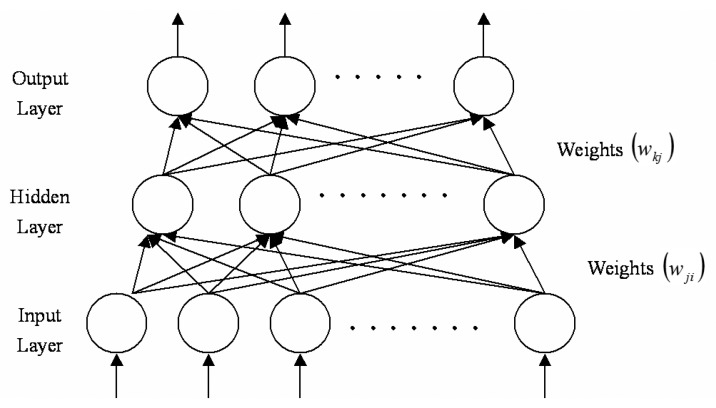
The architecture of a three layer backpropagation neural network.

Back-propagation network (BPN) architecture consists of a three-layered network unit: input, intermediate, and output unit; its architecture is shown in Figure 2, where *w_ji_* are the weights between the hidden layer and the input layer, and *w_kj_* are the weights between the output layer and the hidden layer. The basic element of the neural network is the processing node, which performs two functions: first, it sums the values of inputs and then passes through an arbitrary activation function to produce the node's output value. The activation function used in this study was a sigmoid function, defined as:

(2) $$f(net)=\frac{1}{1-e^{-\text{net}}}$$

where *net* is the sum of the weighted inputs to the processing node.

For the training stage of supervised pattern recognition, the network weights are adjusted in an iterative training procedure called back-propagation [49]. It is a gradient descent method of optimization executed iteratively, with implicit bounds on the distance moved in the search direction of weight space. This is achieved by incorporating a learning rate and a momentum term in the model. The BPN structure is simple, but it can be used to perform many classification or prediction tasks. In this study, we are using the back-propagation neural network for the estimation of water quality from the hyperspectral data. The inputs of the neural network are the reflectance, measured between 400 and 920 nm with 30 nm intervals and the output layer, which has only one node whose output value is the estimation of water quality.

## 5. Results

### 5.1. Spectral Characteristics of Water Quality

Table 1 shows the characteristics of the water samples collected from the Wu River basin. The TSS measured at the river (*n* = 160) ranged from 68 to 3442 mg·L^−1^ and the mean value of TSS was recorded as 416 mg·L^−1^. However, only 9% of the samples (*n* = 14) had TSS values less than 250 mg·L^−1^. The values of Chl-a were in the range from N.D. to 9.6 μg·L^−1^, while the COD concentration ranged from N.D. to 94.5 mg·L^−1^.

**Figure 3.**
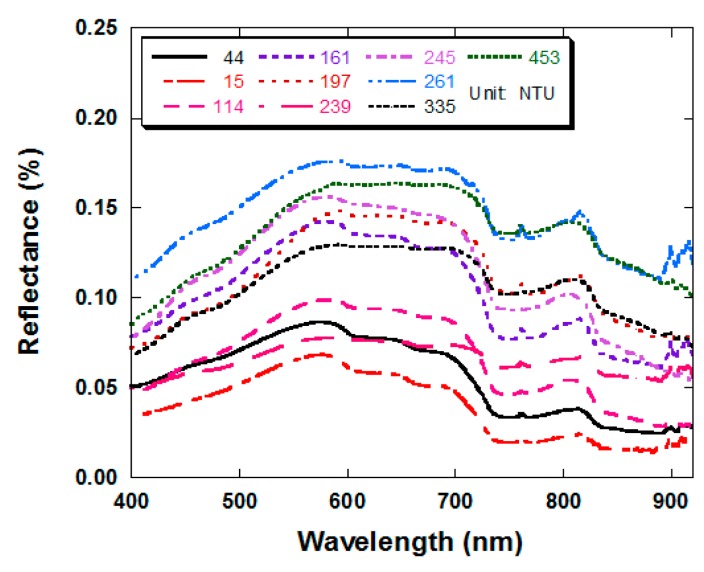
Representative reflectance spectra of surface water with varying concentration of turbidity in the Wu River basin, Taiwan.

Table 2 shows the results of the Pearson correlation between various water quality parameters. The correlation of turbidity with TSS was 0.766, which implies that turbidity is significantly related to TSS in the Wu River system. However, the correction coefficients between TSS and Chl-a or pH were −0.212 and −0.113, respectively. On the other hand, COD has a correction coefficient of 0.494 with TSS and 0.239 with turbidity. The spectra collected across the Wu River basin thus revealed the dominant influence of TSS and turbidity on the characteristics of the reflectance spectra. Figure 3 shows the reflectance spectra for various turbidity levels. Based on the results, it was found that turbidity correlated positively across the entire spectrum in agreement with results reported by other researchers [24,50,51]. The spectral features included: (1) maximum reflectivity between 550 and 700 nm, caused by a local minimum in the combined absorption effects of Colored Dissolved Organic Matter (CDOM) and Chl-a absorption; and (2) over 700 nm at which the water molecules absorb radiation strongly in the infrared range of the spectrum.

The absorption coefficient increases exponentially with increase in wavelength until 900 nm; and signals became weak beyond 920 nm wavelength; (3) Minimum reflectivity is at 900 nm; (4) Considerable fluctuations were observed in the profile below 400 nm and above 930 nm due to noise interference. This result is consistent with a previous investigation [34]. Tolk *et al.* [52] investigated how bright and dark bottoms can influence the reflectivity in relation to varying suspended sediment concentrations measured at 663 and 670 nm using fluorescence at 663 nm emission wavelength and 670 nm excitation wavelength. A turbidimeter was used to measure turbidity at all wavelengths above 550 nm as a result of increase in SSC, and peak reflectance shifted to longer wavelengths. Above 580 nm, the rate of increase in reflectance with SSC addition appeared in an order, particularly between 740 and 900 nm, indicating a strong association between SSC and reflectance at these wavelengths.

As turbidity increased from 10 to 600 NTU, the reflectance increased uniformly for 400 and 900 nm wavelengths regions. However, at less than 400 nm and more than 920 nm wavelengths, visual separation of spectral curves was more difficult. The profiles of spectral reflectance against the TSS concentration were similar to the turbidity levels. At 600 NTU or above levels of turbidity the spectral became somewhat irregular or were virtually indistinguishable for the most part, suggesting that the relationship was becoming non-linear (data not shown). This evidence may indicate that one should be cautious in the use of linear regression models for estimating turbidity levels above 600 NTU from spectral reflectance.

### 5.2. Correlating Reflectance with TSS and Turbidity

**Figure 4.**
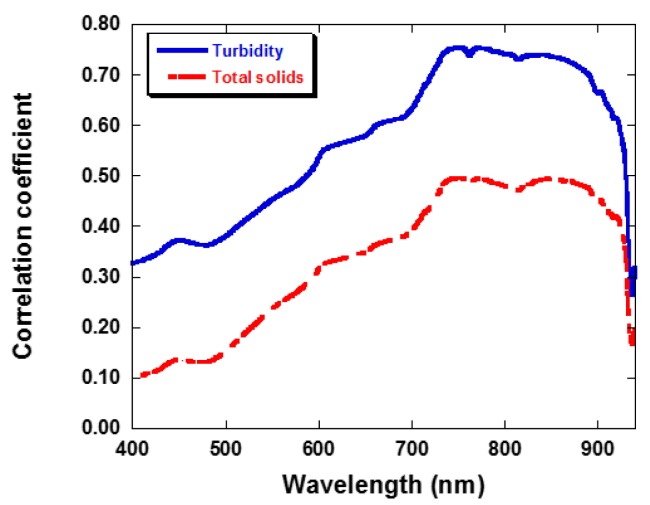
Examples of the change in the Pearson's product moment correlation coefficient with wavelength of water quality variables.

Statistical techniques are the most commonly used approaches to derive a correlation between spectral data and TSS concentrations [26,50,53]. Previous investigations showed that the spectral data best predicted TSS variation with water conditions [23]. To examine the relationships between TSS/turbidity concentrations and spectral data, a Pearson correlation analysis and a regression analysis were applied separately on the data under various water conditions. The Pearson correlation coefficient (r) was calculated to examine the strength of the relationship between TSS and turbidity with corresponding to the spectral reflectance measured at each spectroradiometer channel between 400 and 920 nm (Figure 4). Thus, total 520 correlation coefficients were computed. Results ranged from 0.33 to 0.75 and 0.10 to 0.49 for turbidity and TSS, respectively. Apparently, wavelength has a strong effect on the Pearson correction coefficient.

The correlation coefficient between the spectral reflectance and TSS/turbidity concentration increased as the wavelength increased from 400 to 750 nm. The correction coefficients almost remained constant from 750 to 900 nm and then a steep declining trend was seen at wavelengths above 920 nm. The highest correlation coefficients between the water quality parameters and the spectral reflectance along with their *r* values showed that near IR (750–900 nm) can give the best relationship for the prediction of TSS and turbidity values. The results agreed well with other researchers [7,54,55]. Bhargava and Mariam [7] demonstrated that 700 to 900 nm was the optimal wavelength range for measuring suspended sediment concentrations. Suspended sediment in water had greater absorption of radiation at longer wavelengths [56]. Ma and Dai [55] measured the field spectral reflectance in Taihu Lake of China using a spectroradiometer. It was concluded that the reflectivity in the range from 760 to 1100 nm plays an important role in estimating TSS concentrations. In contrast, no significant relationship was found between Chl-a and spectral reflectance for the Wu River basin. This was partially due to the high TSS and turbidity levels and relatively low Chl-a concentrations. The results are consistent with previous investigations [20,23,54]. A near-infrared band would be well suited to determine the total suspended matter (TSM) concentration in lake waters, dominated with suspended sediment in presence of relatively low phytoplankton algae concentrations [20,23,57]. Han and Rundquist [54] conducted a laboratory experiment to investigate the relationship between surface spectral reflectance and SSC and found that from 700 to 900 nm range is an optimal wavelength for measuring SSC. A consistent increasing trend has been observed for the correlation coefficients with increasing wavelength as well as the concentration of TSS and turbidity. The values of the correction coefficient for TSS were lower than turbidity. The mean difference of correlation coefficient between TSS and turbidity was 0.24 (standard deviation = 0.01) for 400 and 920 nm wavelength range. Measurements of nephelometric turbidity were included in the analyses as an optical method related to the scattering and absorbance characteristics of the water. It differs from the TSS concentration, for example, which is a measure of the weight of inorganic particulate suspended in the water column. This low coefficient indicated that TSS was not the only variable to control the turbidity in the river water as measured by the optical technique. The COD was the other variable controlling the turbidity levels. Even though, the COD fraction clearly has less influence on the optical properties of the water, when compared with TSS.

### 5.3. Comparing Predictive Models for Turbidity and Total Solids

#### 5.3.1. Regression Analysis

Regression analysis was performed separately on the two datasets for training and testing the algorithm. The independent variables were the spectral reflectance measured at each channel between 400 and 920 nm, while the dependent variables were the corresponding concentrations of TSS and turbidity. The correlations between the concentrations of TSS, turbidity and spectral reflectance were performed. Results showed that the correlation coefficients of single variables were poor for characterizing the relationship between the water quality parameters and the spectral reflectance (data not shown). Therefore, to find the best combination for comparison, MR with the forward stepwise procedure was established for the many different combinations of bands. To indicate the significance of the regression models, several parameters, including the coefficient of determination (R^2^) and the standard error of estimate (SEE) was used here.

**Figure 5.**
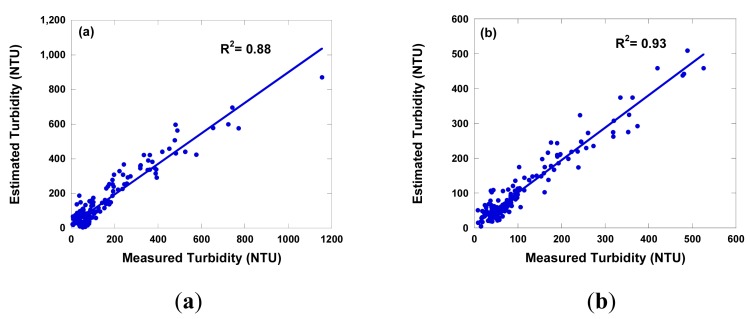
The correction between measured turbidity from *in situ* sampling and estimated turbidity derived from field spectral using multiple regression equation. (**a**) all data; (**b**) exclude above 600 NTU.

Table 3 shows the MR summary for turbidity statistical prediction model with R^2^ value and SEE. Based on the results, the most successful combination for turbidity was used as a linear equation including blue band and the near IR band. Adding four more bands did produce a better fit in this case and generally increased the R^2^ values slightly. A significant relationship (R^2^ more than 0.8) was observed, and the SEE was 80. Figure 5a shows the measured plot and the best estimated concentration of turbidity. The R^2^ and SEE from the regression model for turbidity were 0.88 and 62, respectively. These algorithms differ in their choice of bands from other studies [45,47], this was expected because the different types of sediments and the other water parameters can affect the optical properties of the water at different geographical sites.

**Figure 6.**
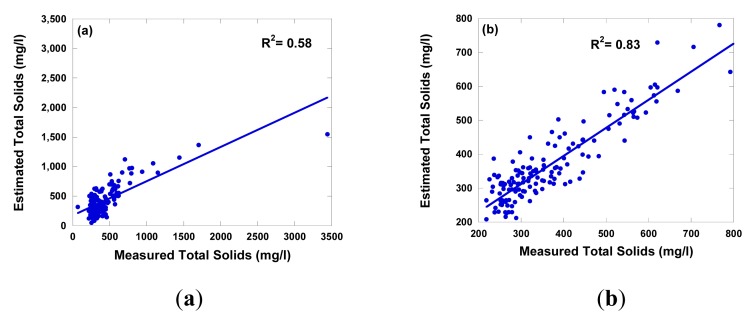
The relationship between measured TS from *in situ* sampling and estimated turbidity derived from field spectral using multiple regression equation. (**a**) all data; (**b**) exclude above 800 mg·L^−1^.

Figure 6a shows the measured and estimated concentrations of TSS. The overall results of TSS (R^2^ = 0.58, SEE = 211) was worse than those of turbidity (R^2^ = 0.88, SEE = 62). This was also expected, since as mentioned above the TSS has a lower correlation with spectral reflectance than turbidity because turbidity is an optical measurement of water quality and differs from TSS which is a measurement of weight of inorganic/organic particles in the water column. A comparison of TSS and turbidity concentrations were estimated using the prediction model with the real TSS and turbidity concentrations measured in the field. The data showed that, in general, the estimated TSS and turbidity concentrations were low at high concentrations. This result, combined with the fact the slope of the regression lines is always less than unity (Figures 5a and 6a), which indicates that the equations underestimate at high concentrations. The plot of estimated versus measured TSS and turbidity concentrations below 800 mg·L^−1^ and 600 NTU were generally scattered around the 1:1 line. The largest deviations from the 1:1 line was observed usually for TSS and turbidity concentrations above 800 mg·L^−1^ and 600 NTU, respectively. The results demonstrated that the accuracy of turbidity and TSS estimation applying regression model using TSS and turbidity concentrations below 800 mg·L^−1^ and 600 NTU (R^2^ = 0.93 for turbidity and R^2^ = 0.83 for TSS) is much better than those using the whole dataset (Figure 5b and 6b). A number of studies have indicated that the amount of solar radiation reflected from surface water reaches at maximum as suspended sediment concentrations in the surface water increases [3,48,54]. Thus, a TSS and turbidity prediction model as used in this study would tend to underestimate at high concentrations of TSS and turbidity. This evidence may suggest that one should be cautious in using a TSS and turbidity prediction model for estimating the TSS and turbidity levels above 800 mg·L^−1^ and 600 NTU, respectively from spectral reflectance. The validation of the regression model was carried out using the water samples (*n* = 10) taking from the other river basin, *i.e.*, the Maoiuo River. The results were satisfactory (R^2^ = 0.82) for turbidity, however, poor for TSS (R^2^ = 0.45). There are several reasons for the poor estimated TSS relations, including the various types and grain sizes of sediment, [58,59]. Gin *et al.* [58] studied the spectral reflectance of water containing concentrations of organic and inorganic sediments isolated under controlled experimental conditions and natural sunlight. The results showed that various types of sediments can affect these spectral profiles. Small sized particles led to greater scattering, and therefore an overall increase in spectral reflectance.

#### 5.3.2. Artificial Neural Network

To compare the regression analysis with standard methods of water quality estimation, a neural network study was also performed on the dataset. The neural network used in this study was a multilayer and feed-forward type that employs back-propagation of error. The neural network was trained several times for both TSS and turbidity. Some combinations resulted in the network becoming trapped in a local minimum and never converging to an acceptable error level. After several trials with different initial weights, the combination of weights with the best performance was retrained for the use of entire spectral reflectance.

Before training, the input and target output training sets were scaled between 0 and 1. The number of data used in the hidden nodes was half that of the input node. Table 2 gives the R^2^ values determined by the artificial neural network method. The results showed that turbidity level was correlated better to reflectance than TSS concentration in network analysis. The coefficient of determination was 0.87 and 0.66 for turbidity and TSS, respectively. This was expected as indicated in Figure 4, where TSS had a lower correlation with spectral reflectance than turbidity over the entire spectrum.

The validation of the ANN was carried out using the water samples (*n* = 10) taking from the Maoiuo River. The statistics of turbidity estimation from Maoiuo River data set using ANN approaches, as well as the comparison with those from Wu River basin data set, is presented in Table 3. The results show that the turbidity is satisfactory (R^2^ = 0.86) as compared to TSS. The R^2^ from ANN for the validation data set (Maoiuo River) was 0.36, and 0.66 for training data set (Wu River data set). It was expected that the TSS prediction model would be affected by the sediment type, mineral composition, grain size as well as optical properties of the suspended solid presented in inland water.

### 5.4. Compares with Regression Analysis and Neural Network

Table 4 shows the comparison of statistics between the results of MR and ANN for the estimation of both turbidity and TSS. The ANN approach can improve the TSS retrieval using the MR approach. These results demonstrate that the accuracy of TSS estimation of applying ANN (R^2^ = 0.66) is better than with the MR approach (R^2^ = 0.58). This was already expected, due to the nonlinear nature of the transfer function. Keiner and Yan [8] used Landsat Thematic Map data which was combined with surface measurements of water quality (including SSC) to map the conditions of Delaware Bay. The R^2^ from neural network for SSC were 0.54 as compared to 0.97 for the regression analysis. The analysis showed that the network is able to model the nonlinear transfer function better than traditional regression analysis, though regression analysis would still be an easier tool for transfer functions that are linear in behavior, but their nonlinearities are well known.

## 6. Conclusions

Results clearly indicated that a hyperspectral sensor can be a useful tool for water quality monitoring in terms of both TSS and turbidity concentrations for the sites which contain high levels of turbidity or TSS concentrations. The traditional methods of river water monitoring are time consuming, costly, and sometimes impractical to apply on a large scale. Hence, the present study can provide relatively a better option to monitor the river water characteristics using hyperspectral sensing techniques. The following findings were obtained:

1. Field sampling demonstrated significant positive correlations between TSS concentration/turbidity levels and spectral reflectance.
2. The best wavelength for monitoring of turbidity level and TSS concentrations was obtained using the hyperspectral sensor between 700 and 900 nm.
3. Non-linearity of the relationship between TSS and turbidity levels and surface spectral reflectance was observed for TSS and turbidity above than 800 mg·L^−1^ TSS and 600 NTU level.
4. The correlation coefficient between turbidity levels and reflectance was greater than TSS concentration and reflectance as well.
5. ANN has shown its usefulness in the modeling of transfer function between the water quality parameters like TSS and turbidity concentrations and the received radiance of a spectroradiometer. The ANN can model the nonlinear transfer function better than MR analysis, and MR analysis would be a better tool for transfer functions which are linear in behavior.
6. The method outlined in this paper could be applied to other inland waters, but the specific coefficients of the model may vary as a consequence of optical characteristics of the TSM, depending on the sediment type and particle size distribution as well.

## Figures and Tables

**Table 1.** Water quality parameters of Wu river samples.

	**TSS (mg/L)**	**Turbidity (NTU)**	**COD (mg/L)**	**Chl (μg/L)**	**pH**
Minimum	68	7	N.D.	N.D.	6.8
Maximum	3442	1156	94	10	8.1
Mean	416	150	19	2	7.6
Std. Deviation	319	176	13	2	0.3

N.D. means not detected.

**Table 2.** Pearson correlation matrix of water quality parameters.

	**TSS**	**Turbidity**	**COD**	**Chl**	**pH**
TSS	1.000	0.766 (**)	0.494 (**)	−0.212	−0.113
Turbidity	-	1.000	0.239 (*)	−0.198	−0.081
COD	-	-	1.000	−0.280	−0.009
Chl	-	-	-	1.000	0.055
pH	-	-	-	-	1.000

** Correction is significant at the 0.01 level (2-tailed);

* Correction is significant at the 0.05 level (2-tailed).

**Table 3.** Summary of MR and SEE model values for the statistical prediction of turbidity.

**No. x_n_(wavelength)**	**Coefficient**											**R^2^**	**SEE**

	**a_0_**	**a_1_(754)**	**a_2_(401)**	**a_3_(809)**	**a_4_(920)**	**a_5_(820)**	**a_6_(914)**	**a_7_(453)**	**a_8_(736)**	**a_9_(882)**	**a_10_(453)**		
1	82	4519	−3977	-	-	-	-	-	-	-	-	0.69	99
2	133	22,159	−4670	−16,133	-	-	-	-	-	-	-	0.77	86
3	110	25,831	−4116	−18,072	−2161	-	-	-	-	-	-	0.80	80
4	163	30,673	−3490	−60,192	−6685	40,568	-	-	-	-	-	0.84	72
5	153	32,263	−3458	−56,670	−14,018	34,687	8028	-	-	-	-	0.85	70
6	155	34,381	−7228	−66,092	−14,058	42,002	7991	3284	-	-	-	0.86	69
7	159	49,988	−7703	−77,437	−14,956	59,245	7061	4781	−20,732	-	-	0.86	67
8	140	76,997	−8404	−94,947	−9741	77,412	6123	5548	−41,024	−11,940	-	0.87	65
9	157	93,231	−8641	−97,982	−9717	78,788	3823	5821	−52,633	−32,005	19,381	0.88	63

**Table 4.** Comparison between the results of regression and neural network.

**Analytical Method**		**Total Solids**		**Turbidity**	
	
		**Regression**	**Neural Network**	**Regression**	**Neural Network**
R^2^	Wu River basin	0.58	0.66	0.88	0.87
	Maoiuo River	0.45	0.36	0.82	0.86
